# Puerarin inhibits the osteoclastogenesis by inhibiting RANKL-dependent and –independent autophagic responses

**DOI:** 10.1186/s12906-019-2691-5

**Published:** 2019-10-15

**Authors:** Guoyou Zhang, Yu Wang, Guoke Tang, Yuanzheng Ma

**Affiliations:** 1 Department of Orthopedics, Southern Medical University, Guangzhou, 510515 Guangdong China; 2 Department of Orthopaedics, The 8th medical center of chinese PLA general hospital, No. 17 Heishanhu Road, Haidian District, Beijing, 100091 China; 3 Department of Orthopaedics, Inner Mongolia Tongliao City Hospital, Tongliao, 028000 Inner Mongolia China; 4 Department of Orthopaedics, Chifeng Hospital, Chifeng, 024000 Inner Mongolia China; 5 Department of Orthopaedics, The affiliated Zhuzhou hopital XiangYa medical college CSU, Zhuzhou, 412000 Hunan China

**Keywords:** Puerarin, Osteoclast, Autophagy, BECN1, Osteoporosis

## Abstract

**Background:**

Puerarin exerts therapeutic effect on osteoporosis due to its inhibitory effect on the formation of osteoclasts. Puerarin is also widely established as an autophagy inhibitor. The study aimed to investigate the significance of autophagy in Puerarin-treated osteoclast formation.

**Methods:**

Osteoclast precursors (OCPs) derived from bone marrow-derived macrophages (BMMs) were treated with Puerarin along with RANKL or without RANKL, and then the autophagic parameters of OCPs (including autophagic proteins, LC3 transformation, autophagosome or LC3-puncta) were observed through Western Blotting, Transmission Electron Microscopy and Immunofluorescence assays. Next, after using overexpression vectors of autophagic genes (Atg7, Atg5 and BECN1) to alter autophagy activity, OCP proliferation was measured by Ethynyl deoxyuridine (EdU) assays and Cell Counting Kit-8 (CCK-8) kit, and osteoclast differentiation was assessed by Tartrate-resistant acid phosphatase (TRAP) staining.

**Results:**

The results showed that Puerarin could directly inhibit the autophagy and proliferation of OCPs. Importantly, overexpression of autophagic genes Atg5, Atg7 and BECN1 reversed Puerarin-inhibited OCP autophagy and proliferation. What’s more, RANKL could promote the autography of OCPs, which was recovered by Puerarin treatment. Interestingly, different from single-Puerarin treatment, we found that in the presence of RANKL, only BECN1 overexpression significantly reversed Puerarin-inhibited osteoclast differentiation and OCP autophagy.

**Conclusion:**

In conclusion, Puerarin could inhibit the OCP autophagy in the presence or absence of RANKL, which blocked the OCP proliferation and osteoclast differentiation respectively. Moreover, BECN1 plays an essential role in Puerarin-inhibited osteoclastogenesis. Our study provides potential clue to further complete the intrinsic mechanism of Puerarin in treating osteoporosis.

## Background

*Pueraria lobata* is a leguminous plant in China, which is widely used in the treatment of cardiovascular diseases, diabetes, osteonecrosis and neurodegeneration [1]. As an extract from *Pueraria lobate*, Puerarin is a phytoestrogen with significant bone-protective effect. Its therapeutic effect has been broadly reported in the treatment of osteoporosis. Cho et al. [2] found that the decrease in the femoral bone density of ovariectomized mice was inhibited after feeding Puerarin-containing diet for 4 weeks. Another study found that Puerarin could alleviate streptozotocin-induced osteoporosis in rats via HDAC1/HDAC3 signaling [3]. In addition, Puerarin could also prevent bone loss in castrated male rats [4]. The inhibitory effect of Puerarin on osteoclasts matters a lot in its treatment of bone loss. Pueraria extracts are known to inhibit RANKL-stimulated osteoclastogenesis in the dose-dependent manner and to reduce bone resorption activity of osteoclasts [5]. Puerarin also reduced the formation of mature osteoclasts in RANKL-induced RAW264.7 cells [6]. In addition, Puerarin can also prevent lipopolysaccharide-stimulated osteoclastogenesis and bone loss [7]. However, the mechanism regarding the effect of Puerarin on inhibiting the osteoclastogenesis remains unclear.

Previous studies disclosed that the protective autophagy exerts an indispensable effect on the osteoclast formation as well as bone absorption activity of osteoclast [8–10]. In addition, a considerable number of studies suggested that Puerarin could regulate the autophagic activity. Some reports have shown that Puerarin upregulates autophagy [11–13], but some studies showed that it inhibits autophagy [14–16]. He et al. [14] suggested that Puerarin had neuroprotective effect against cerebral ischemia, which was related to the decrease of autophagic activity in neurons after its intervention. It was also reported that Puerarin could prevent rat brain from ischemia/reperfusion injury through repressing the autophagic response [15]. In addition, Puerarin pretreatment reduced the hypoxia/reoxygenation injury via inhibiting the Akt-autophagy signaling in the myocardium [16]. Therefore, whether the effect of Puerarin on inhibiting the osteoclastogenesis is by mediating the change of autophagic activity is worth further exploring.

Osteoclastogenesis consists of the proliferation and differentiation of OCPs. RANKL is a key inducing factor in osteoclast differentiation. Autophagy not only plays an important role in OCP proliferation [17], but also regulates OCP differentiation under RANKL intervention [8, 9]. Thus, we can clarify the overall significance of autophagy in Puerarin-treated osteoclastogenesis by observing the effects of Puerarin on the autophagic activity of OCPs in the presence and absence of RANKL, respectively. During the autophagy response, a cytosolic form of LC3 (LC3I) forms membrane-bound LC3 (LC3II) by conjugating to phosphatidyl inositol. Thus, LC3 transformation and LC3 puncta are pivotal parameters for observing autophagic activity [18], including the osteoclastogenesis [8, 10, 19, 20]. On the one hand, as important autophagy parameters, LC3 conversion rate and LC3 puncta number were upregulated by RANKL in OCP [8, 19]; On the other hand, LC3 plays a significant role in the osteoclastogenesis. In this study, the detection of autophagic activity was focused on LC3-related indicators [10].

This study showed a role of Puerarin in inhibiting the OCP autophagy in the absence or presence of RANKL, which contributed to the reduction in OCP proliferation or OCP differentiation, respectively. Therefore, by clarifying the significance of Puerarin in the OCP autophagy, the present study revealed an autophagic mechanism underlying Puerarin-treated osteoclastogenesis for the first time.

## Methods

### Reagents

Recombinant M-CSF and RANKL were purchased from Peprotech (Rocky Hill, NJ, USA). Puerarin, E64d, Pepstatin A (PEPS A) and TRAP staining kit were obtained from Sigma-Aldrich (St. Louis, MO, USA). Rabbit LC3B, Atg5, Atg7, Beclin1, and β-actin antibodies were purchased from Cell Signaling Technology (Beverly, MA, USA). Cell Counting Kit-8 (CCK-8) kit was obtained from Dojindo (Shanghai, China). The EdU kit was purchased from Roche (Mannheim, Germany). The SYBR Premix Ex TaqTM kit was from TakaRa (Tokyo, Japan). After dissolving in 1% BSA, different working concentration of Puerarin (0, 10, 25, 50 μM) were prepared by complete α-minimum Eagle’s medium (α-MEM).

### Isolation and induction of OCPs

4–8 week old littermate C57BL/6 J female mice were purchased from Slaccas Experimental Animal Centre (Shanghai, China). The mice were housed in a common environment in which the room temperature was 20~30 °C and the humidity 60~80% and fed a general laboratory diet. The mice were sacrificed by cervical dislocation, and the tibia from mice were flushed using α-MEM without serum. Bone marrow cells were incubated with a-MEM containing 10% FBS, penicillin (100 U/ml) and streptomycin (100 mg/ml) for 24 h. Non-adherent cells were collected as Bone marrow-derived macrophages (BMMs). BMMs were induced to OCPs (adherent cells) after treatment with M-CSF (20 ng/ml) for 3 days as previously described [21, 22]. Cells were cultured in the humidified atmosphere at 37 °C and containing 5% CO_2_. The experimental protocols were approved by the Institutional Animal Care and Use Committee of Southern Medical University (No.44002100017774). To further elucidate the direct effect of Puerarin on the expression of autophagic proteins, cells were treated with Puerarin at different concentrations for 6 h without RANKL.

### Osteoclast differentiation assay

OCPs (1 × 10^5^/well) were incubated in 24-well plate in α-MEM involving M-CSF (20 ng/ml) plus RANKL (100 ng/ml) supplemented with other relevant treatment for 5 days to induce mature osteoclasts. TRAP staining-positive multinucleate cells (having more than three nuclei) were regarded as the differentiated osteoclasts.

### Cell proliferation assay

Cell proliferation was evaluated using EdU assays and CCK-8 kit. In EdU assays, cell proliferation was quantified by observing EdU-positive cells, and in CCK-8 analysis, cell proliferation was measured by observing relative cell number. For EdU assays, OCPs (1 × 10^5^/well) were cultured in 6-well plate, and treated with indicated treatment. Then, EdU assays were performed by using the EdU kit in accordance with the instruments. The results were collected and quantified under the Zeiss Photomicroscope (Carl Zeiss, Oberkochen, Germany) on the basis of at least ten random fields. For CCK-8 analysis, OCPs were incubate in 96-well plate at a density of 1 × 10^4^/well, and then treated with different interventions. Next, cells were treated with the CCK-8 reagents for 1 h. Ultimately, the optical density at 450 nm (OD450) was observed by using Varioskan Flash reader (Thermo, MA, USA).

### Lentivirus infection

Lentivirus encoding Atg5, Atg7 or BECN1 (including the corresponding control vector) were constructed by homologous recombination between expression vector (EX-Puro-Lv105) and cDNA in 293 cells using the construction kit (GeneCopoeia, MD, USA) as previously described [23]. After 2 days, supernatants were collected and OCPs were incubated in the viral fluid containing 8 μg/ml polybrene at MOI 10 for 2d. Transduced cells were selected by puromycin (7.5 μg/ml). The expression of viral genes was observed using qRT-PCR.

### Western blotting (WB) analysis

The whole-cell lysate protein from cells (about 1 × 10^6^/well) with indicated interventions in 6-well plates were prepared. Lysates were packed into 10% SDS-PAGE gels and polyvinylidene fluoride (PVDF) membranes were incubated with the antibodies against Atg5, Atg7, Beclin1, and LC3B, and β-actin after trarsmembrane. Horseradish peroxidase-linked secondary antibodies were used as secondary antibodies. Bands were visualized using a chemiluminescence system (Amersham Image 600, General Electric, MA, USA).

### Quantitative real-time PCR (qRT-PCR)

The total RNA was extract and purified by the TRIzol method. Synthesis of cDNA and real-time quantitative PCR (qRT-PCR) measurements were performed as described previously [21]. The pre-designed primer sequences for qRT-PCR analysis were as following:

Cathepsin K (CTSK): 5′-GGAAGAAGACTCACCAGAAGC-3′ (forward) and 5′-GTC-ATATAGCCGCCTCCACAG-3′ (reverse); Matrix metalloproteinase-9 (MMP-9):5′-CC-TGTGTGTTCCCGTTCATCT-3′ (forward) and 5′-ACCCGAATCTAGTAAGGTCGC-3′ (reverse); TRAP: 5′-GCTGGAAACCATGATCACCT-3′ (forward) and 5′-TTGAGCCAGG-ACAGCTGAGT-3′ (reverse); Atg7, 5′-GTTCGCCCCCTTTAATAGTGC-3′ (forward) and 5′-TGAACTCCAACGTCAAGCGG-3′ (reverse); Atg5, 5′-ATGCGGTTGAGG-CTCACTTTA-3′ (forward) and 5′-GGTTGATGGCCCAAAACTGG-3′ (reverse); BECN1: 5′-CTAAGGCAGGCAGGAGGATG-3′ (forward) and 5′-GCTGGCCTCAA-GAGATCCAT − 3′ (reverse); Cyclophillin A: 5′-CGAGCTCTGAGCACTGGAGA-3′ (forward) and 5′-TGG-CGTGTAAAGTCACCACC-3′ (reverse).

qRT-PCR analysis was carried out by SYBR Premix Ex TaqTM kit and using ABI7500 analyzer (Thermo, MA, USA).

### Transmission Electron microscopy analysis

Cells (5 × 10^5^) were incubated on 6-cm dishes as described above, followed by indicated treatments. The preparation of cell slices and subsequent staining were performed according to the protocol (Servicebio, Wuhan, Hubei, China) as previously described [19]. Then, the stained slices were observed under the 7700 transmission electron microscopy (Hitachi, Tokyo, Japan).

### Immunofluorescence assay

Cells were incubated on 6-cm dishes, and stimulated with different reagents. The treated cells (1 × 10^6^/tube) were enriched in flow tubes, and then fixed by using 4% paraformaldehyde (PFA). Following perforation, cells were blocked using 1% bovine serum albumin (BSA), and incubated with the antibody targeting LC3B at 4 °C for 12 h. Then, cells were stained with Cy3-labeled Goat Anti-Rabbit IgG for 1 h. Next, cell suspensions were transferred to the adhesive slide. Thirty minutes later, the suspensions were removed, and the cells were counterstained using DAPI. Ultimately, the cells were observed and recorded under the fluorescence microscope (Olympus IX71, Tokyo, Japan). The cells with more than five LC3-punctas were regarded as LC3-puncta positive cells [24, 25].

### Statistics

The data are presented as mean + SEM. Statistical differences among groups were evaluated with one-way ANOVA analysis. Bonferroni test was used for Post Hoc Multiple Comparisons. Statistical significance was set at *P* < 0.05. The statistical analyses were carried out using SPSS 19.0 software.

## Results

### Puerarin directly inhibited OCP autophagy

**Fig. 1:**
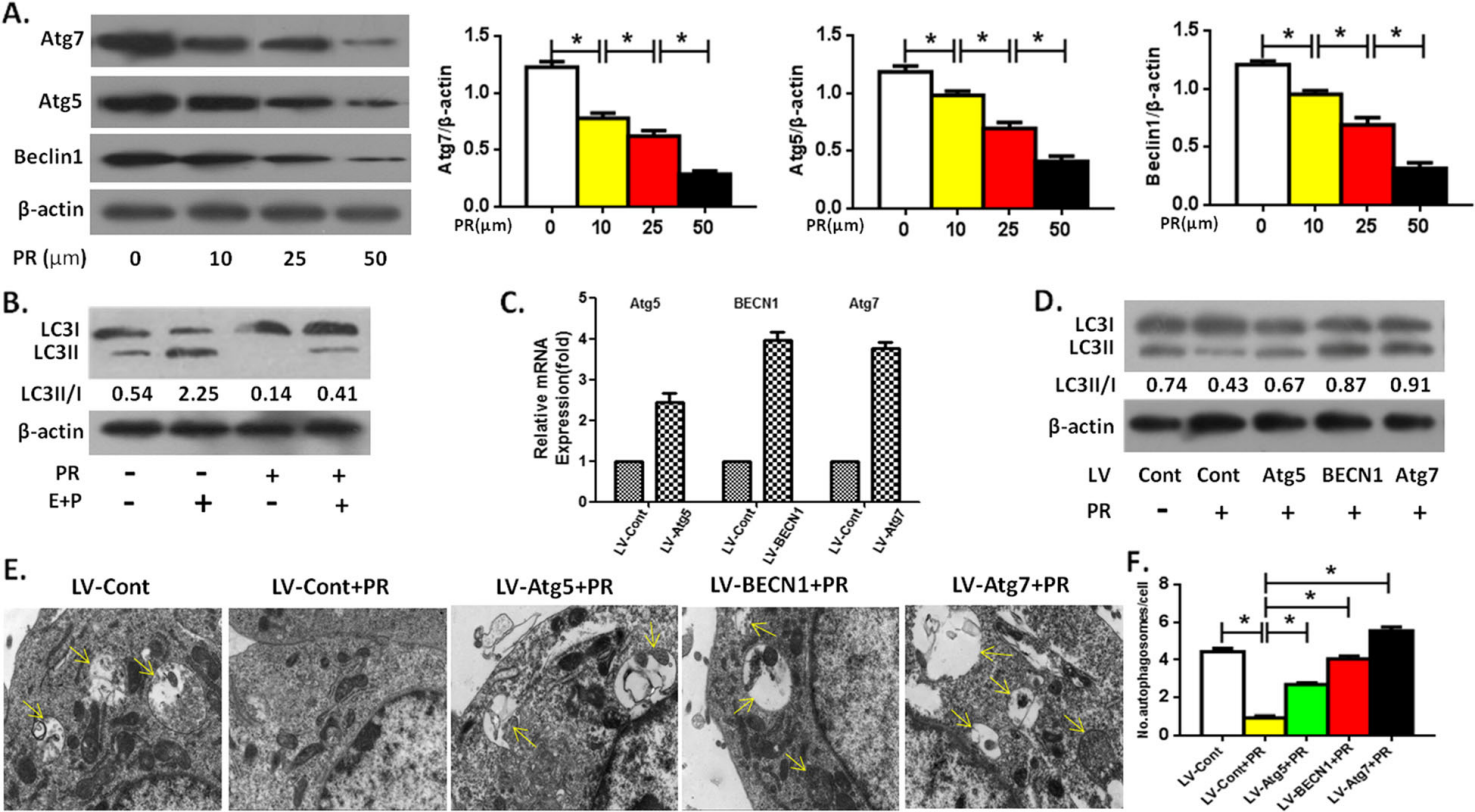
Puerarin directly inhibits OCP autophagy. **a** The protein levels of Atg7, Beclin1 and Atg5 in the OCPs treated with different levels of Puerarin for 6 h without RANKL were measured by Western Blotting. Compared between Group 0 μM and Group 10 μM, Group 10 μM and Group 25 μM, Group 25 μM and Group 50 μM, respectively, *P < 0.05. **b** The ratio of LC3II/I in the OCPs treated with Puerarin (50 μM) in the presence or absence of E64d + PEPS A for 8 h without RANKL. **c** mRNA expression of Atg5, BECN1 or Atg7 in the treated OCPs after infection with lentivirus encoding Atg5, BECN1 or Atg7 (LV- Atg5, LV-BECN1 or LV-Atg5) and the corresponding control vector (LV-Cont). **d** Viruses-transduced OCPs were treated with Puerarin (50 μM) for 6 h without RANKL, and LC3II/I was detected by Western Blotting. **e** The autophagosomes (yellow arrows) in the viruses-transduced OCPs treated with Puerarin (50 μM) for 24 h without RANKL were observed using TEM. Scale bar, 1 μm. **f** Statistical diagrams showed the quantitative results of autophagosomes from E (45 cells from three independent assays). Data are presented as mean ± SEM from three independent experiments. Compared between Group LV-Cont and Group LV-Cont+PR, Group LV-Cont+PR and Groups LV-Atg5/BECN1/Atg7 + PR, respectively, *P < 0.05. PR, Puerarin; Cont, control groups; E, E64D; P, PEPS A

We first observed the direct effect of Puerarin on OCP autophagy without RANKL. The results showed that directly intervened by Puerarin, the expression of autophagic proteins Atg5, Atg7 and Beclin1 in OCPs decreased in a concentration-dependent manner (Fig. 1a). It could be seen that 50 μm Puerarin had the strongest activity in inhibiting the expression of autophagic proteins (Fig. 1a). Thus, we chose this concentration of Puerarin for subsequent experiments. As shown in Fig. 1c-f, 50 μm of Puerarin inhibited the LC3 transformation (defined as LC3II/I) and autophagosome formation in OCPs. Furthermore, LC3II/I was enhanced under the administration of PEPS A plus E64D (Fig. 1b), confirming that autophagy flux under this experimental system was smooth. Nonetheless, the inhibitory effect of Puerarin was reversed when Atg5, Atg7 or Beclin1 gene BECN1 were overexpressed by lentiviral transduction (Fig. 1c-f), and the effect of Atg7 was the most significant (Fig. 1d-f).

### Overexpression of autophagic gene reversed the reduced proliferation of OCPs by Puerarin

**Fig. 2:**
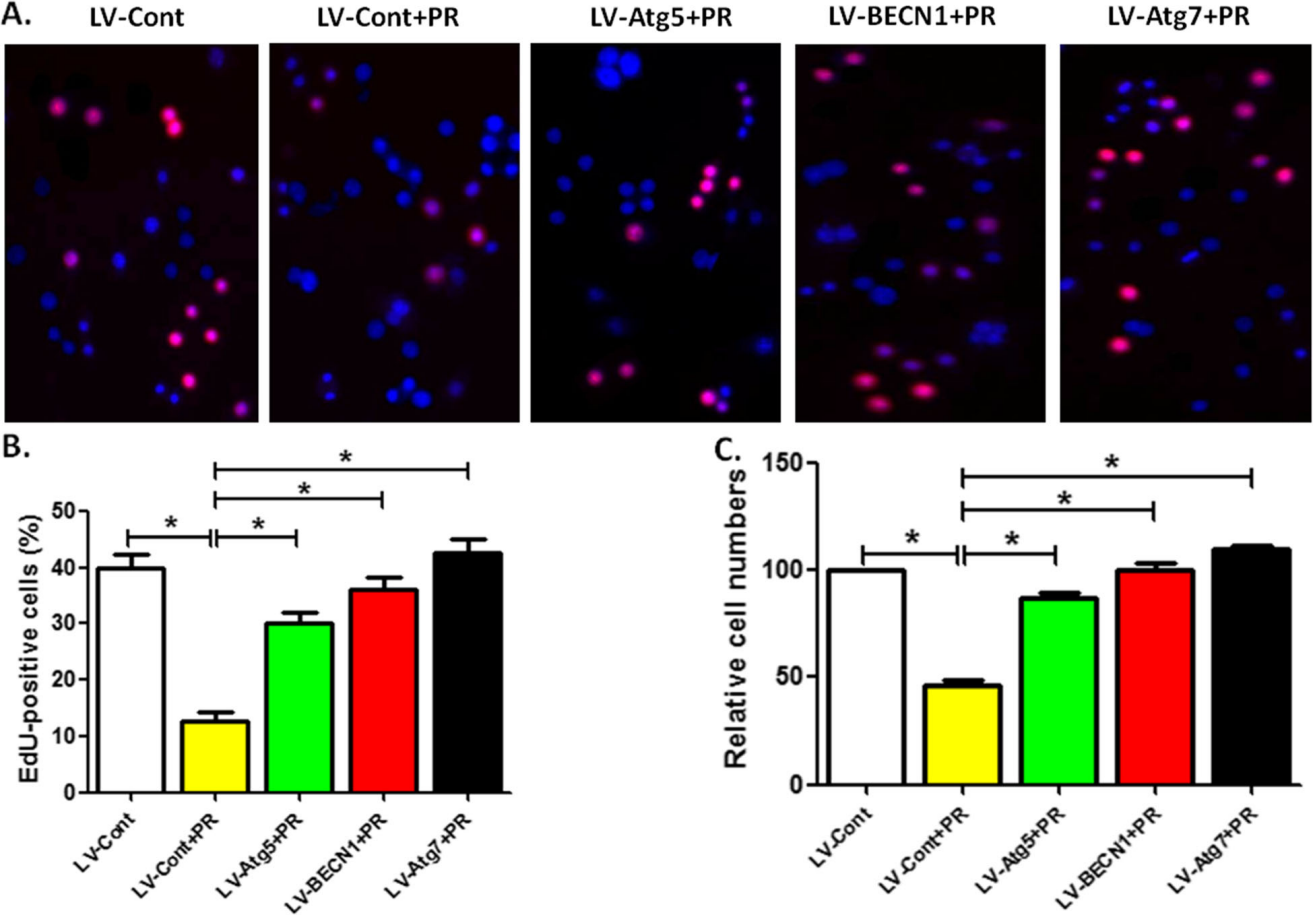
Overexpression of autophagic gene reverses Puerarin-reduced OCP proliferation. **a** The proliferation of viruses-transduced OCPs treated with Puerarin (50 μM) for 24 h was detected using EdU kit. Scale bar, 250 μm. **b** Statistical diagram displayed the percentages of EdU-positive cells in A (50 cells per field, *n* = 10). **c** After treatment as described for (**a**), the OCP proliferation was measured using CCK-8 kit. The group without Puerarin were regarded as the control group (100%). Data are presented as mean ± SEM from three independent experiments. Compared between Group LV-Cont and Group LV-Cont+PR, Group LV-Cont+PR and Groups LV-Atg5/BECN1/Atg7+ PR, respectively, *P < 0.05. PR, Puerarin

Next, we assessed the effect of autophagic regulation on the proliferation of OCPs treated with Puerarin. EdU and CCK-8 assay showed that after 24 h intervention, Puerarin at 50 μm significantly inhibited the relative number of OCPs, which was recovered with the overexpression of Atg5, Atg7 or BECN 1 (Fig. 2a-c). Among them, Atg7 had the strongest recovery effect.

### Puerarin reversed the enhanced effect of RANKL on OCP autophagy

**Fig. 3:**
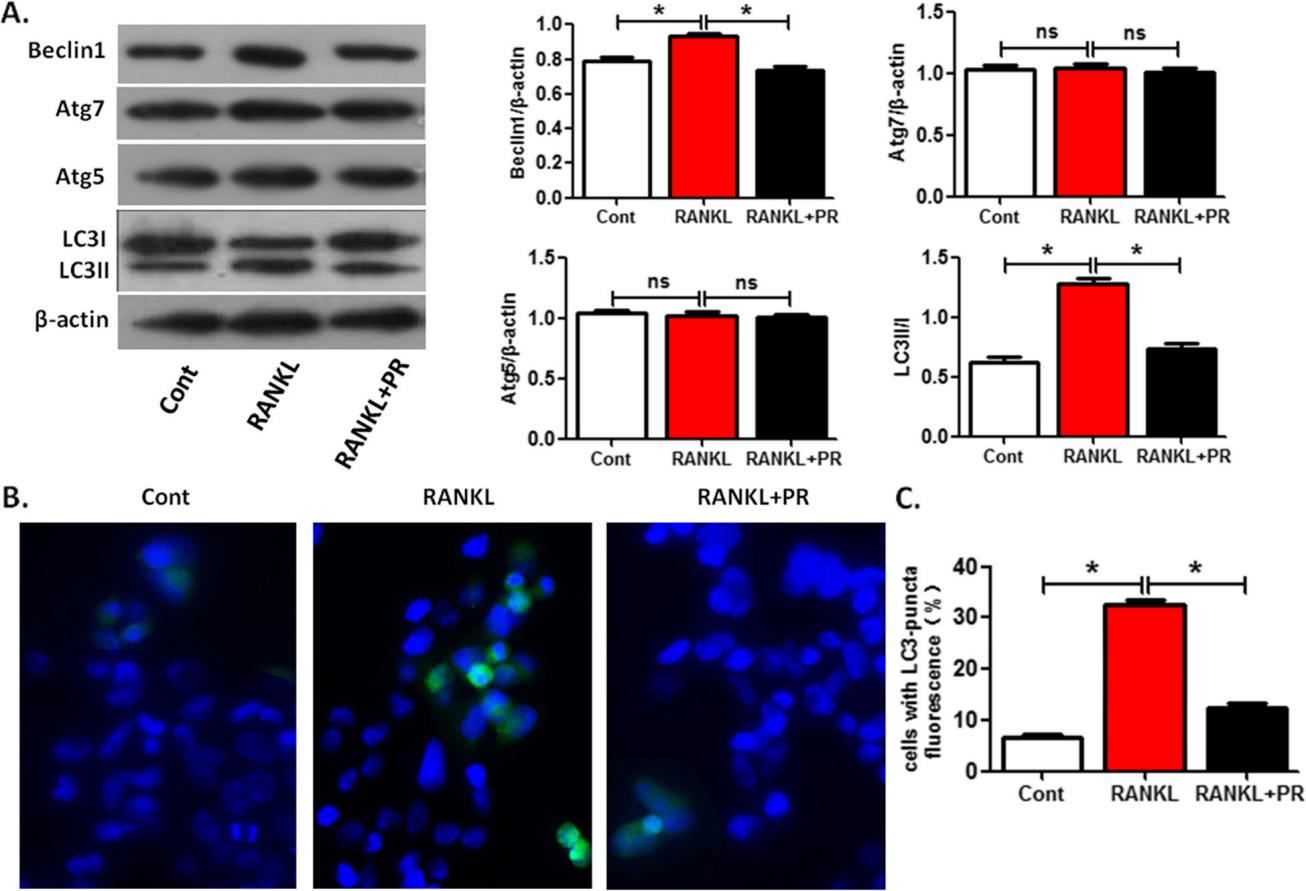
Puerarin reverses RANKL-enhanced OCP autophagy. **a** Following treatment with or without Puerarin (50 μM) for 6 h in the presence of RANKL, the protein level of Atg7, Beclin1 or Atg5, and the ratio of LC3II/I were detected by Western Blotting. **b** After treatment as described for (**a**), LC3-puncta in each group was imaged using the immunofluorescence staining and observed using the fluorescence microscope. Scale bar, 20 μm. **c** Statistical diagrams exhibited the percentages of LC3-puncta positive cells (more than five dots, 50 cells/field, *n* = 5). Data are presented as mean ± SEM from three independent experiments. Compared between control group and RANKL group, RANKL group and RANKL+PR group, respectively, *P < 0.05. ns, no significance; PR, Puerarin; Cont, control groups

The direct effect of Puerarin on regulating the OCP autophagy and subsequent OCP proliferation was documented. It is known that RANKL could promote the autophagy of OCPs [26, 27]. Therefore, we further evaluated the effect of Puerarin on RANKL-regulated OCP autophagy. RANKL upregulated the expression of Beclin1, LC3 transformation and LC3 puncta formation in OCPs, but had no effect on the level of Atg5 and Atg7 (Fig. 3a-c). However, the effect of RANKL on promoting the above autophagic parameters of OCPs decreased with the supplement of 50 μm Puerarin (Fig. 3a-c).

### BECN1 overexpression reversed Puerarin-inhibited OCP autophagy and osteoclast differentiation in the presence of RANKL

**Fig. 4:**
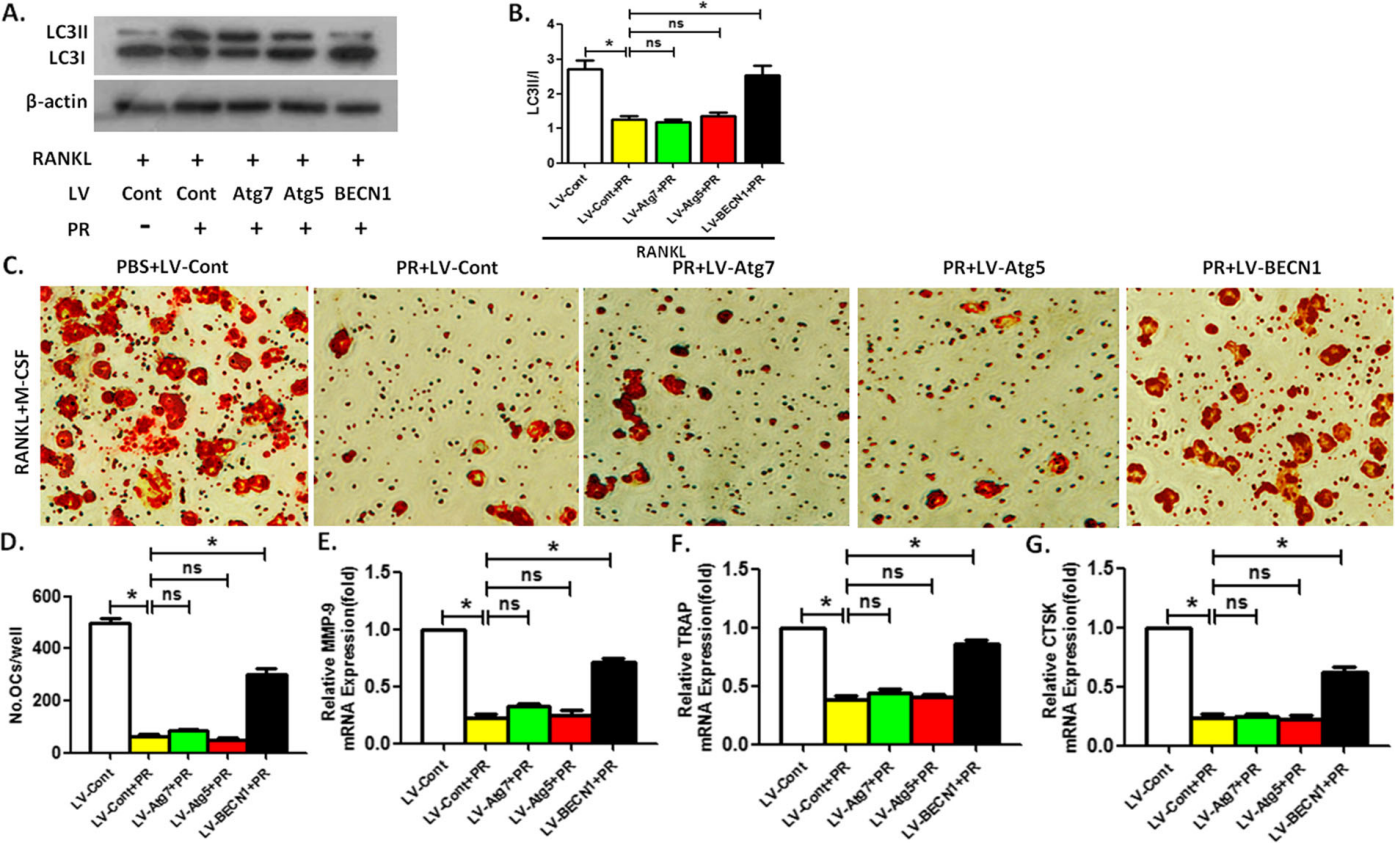
Under RANKL intervention, BECN1 overexpression reverses Puerarin-inhibited OCP autophagy and osteoclast differentiation. **a**-**b** LC3II/I in viruses-transduced OCPs treated with Puerarin (50 μM) along with RANKL for 6 h was detected by Western Blotting. Compared between Group LV-Cont and Group LV-Cont+PR, Group LV-Cont+PR and Groups LV-Atg5/BECN1/Atg7+ PR in the presence of RANKL, respectively, *P < 0.05. **c** Typical TRAP staining of mature osteoclasts derived from viruses-transduced OCPs under the joint intervention of M-CSF, RANKL and Puerarin for 5 days. Scale bar, 100 μm. **d** The quantitative results regarding TRAP^+^ multinucleated cells in C. **e**-**g** mRNA expression of MMP-9, TRAP or CTSK in OCPs treated with indicated treatments. Data are presented as mean ± SEM from three independent experiments. Compared between Group LV-Cont and Group LV-Cont+PR, Group LV-Cont+PR and Groups LV-Atg5/BECN1/Atg7+ PR in the presence of RANKL plus M-CSF, respectively, *P < 0.05. ns, no significance; PR, Puerarin

Next, we observed the effects of the autophagic activity on RANKL-regulated OCP autophagy and osteoclast differentiation in the presence of Puerarin treatment. Under the intervention of RANKL, the LC3 transformation of OCPs inhibited by Puerarin was reversed by BECN1 overexpression, but the overexpression of the other two autophagic genes Atg5 or Atg7 could not affect Puerarin-treated OCP autophagy (Fig. 4a, b). In addition, TRAP staining showed that the mature osteoclasts differentiated from co-inducement of RANKL and M-CSF decreased with the administration of 50 μm Puerarin, which was recovered by BECN1 overexpression (Fig. 4c, d). Overexpression of Atg5 or Atg7 could not reverse the anti-osteoclastogenic effect of Puerarin (Fig. 4c, d). qPCR results revealed that the variation in the mRNA level of the three marker enzymes regarding the osteoclastogenesis was similar to TRAP staining results (Fig. 4e-g).

## Discussion

As a phytoestrogen, Puerarin is known to inhibit the formation of osteoclasts, yet the detailed mechanism remains unclear, which raises an interesting scientific question for investigation. The effect of Puerarin on autophagic response has been verified. Here, we provide the first evidence demonstrating the effect of Puerarin on regulating the autophagic activity of OCPs. Combining up-to-date molecular manipulations and classic pharmacological inventions, our findings revealed an important autophagic mechanism underlying Puerarin-treated osteoclastogenesis.

**Fig. 5:**
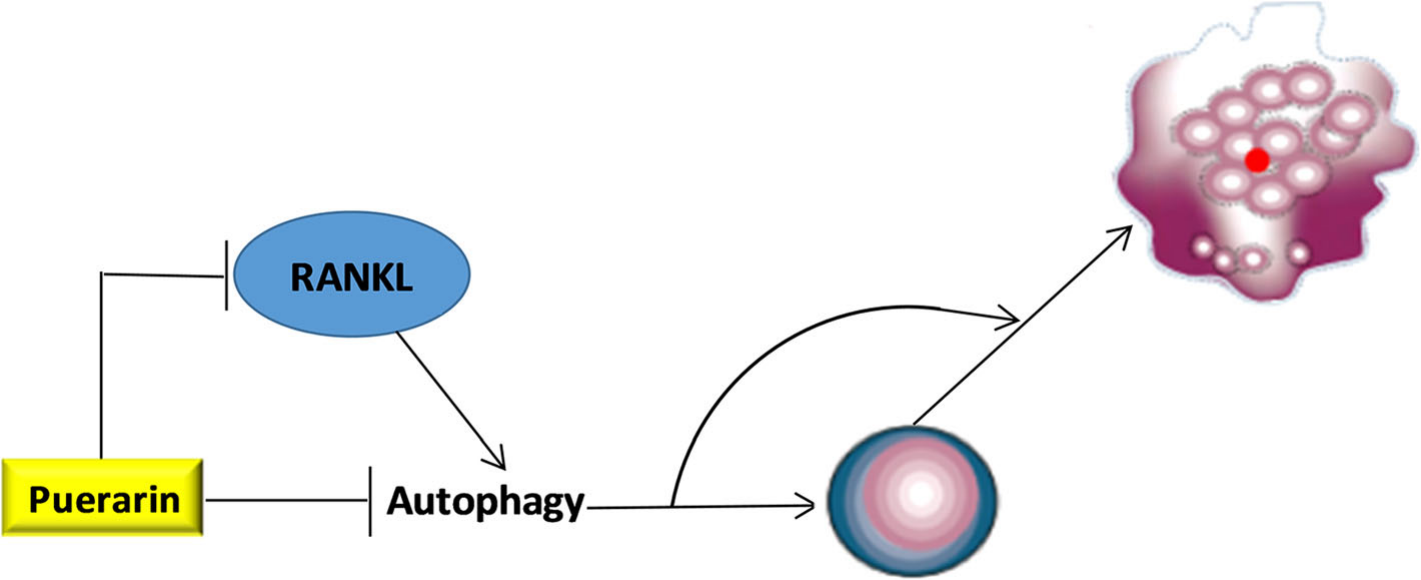
The current working model regarding Puerarin-regulated autophagy during the osteoclastogenesis

Osteoclast formation is known to consist of the proliferation, fusion and differentiation of OCPs [28]. Firstly, we found that Puerarin directly inhibited the autophagy and proliferation of OCPs in the absence of RANKL, which could be reversed by the overexpression of autophagic genes. This suggested that the direct inhibition of Puerarin on autophagy was responsible for its inhibitory effect on the proliferation of OCPs. It should be noted that overexpression of Atg7 had the strongest effect to reverse Puerarin-inhibited OCP autophagy and proliferation, which was inconsistent with the result that under inducement of RANKL, only BECN1 reversed Puerarin-inhibited osteoclast differentiation. The more research is needed to explore the specific contribution of autophagic genes to OCP proliferation, differentiation and subsequent bone resorption under Puerarin treatment in the future. Then, we observed that Puerarin also inhibited the enhancement of RANKL on OCP autophagy. Moreover, BECN1 overexpression reversed the inhibition of Puerarin on OCP autophagy and osteoclast differentiation. Puerarin not only effectively inhibits the expression of RANKL [29–31], but also blocks RANKL-induced osteoclastogenesis [6], suggesting that Puerarin can inhibit RANKL signaling during the osteoclastogenesis. Therefore, Puerarin prevented RANKL-induced osteoclastogenesis by inhibiting the upregulation of RANKL in OCP autophagy. Moreover, Beclin1 could be regarded as a specific autophagic molecule in Puerarin-treated osteoclastogenesis. Moreover, in the presence or absence of RANKL, the autophagic genes that significantly reversed the inhibitory effect of Puerarin were inconsistent. These results suggested that Puerarin might inhibit OCP autophagy via direct inhibition and inhibiting the signal transduction of RANKL, thereby blocking the formation of osteoclasts. Our current model of Puerarin-regulated autophagy during the osteoclastogenesis is illustrated in Fig. 5.

## Conclusion

Puerarin is well accepted as an autophagy regulator and osteoclastogenesis inhibitor. However, the role of autophagy in Puerarin-regulated osteoclastogenesis is still unclear. The present study clarified the potential mechanism regarding osteoclastogenesis treated by Puerarin from the angle of autophagic response. It proved the effect of Puerarin on inhibiting the autophagic activity of OCPs in the absence or presence of RANKL, which is responsible for Puerarin-inhibited proliferation and differentiation of OCPs respectively. This study not only explored the molecular mechanisms of Puerarin-inhibited osteoclastogenesis, which laid the foundation for further investigation, but also presented novel clues for improving the therapeutic strategies of Puerarin in treating osteoporosis. In addition, the role of autophagy in the resorption activity of the osteoclast treated by Puerarin has not been studied, which suggests research gaps to be bridged in future studies.

## Data Availability

The datasets used and analyzed in the current study are available from the corresponding author on reasonable request. All data generated or analyzed during this study are included in this published article.

## Abbreviations

Atg: Autopahgy related gene
BMMs: Bone marrow-derived macrophages
BSA: Bovine serum albumin
Ligand: LC3 Microtubule-associated protein 1 light chain 3
OCPs: Osteoclast precursors
PFA: Paraformaldehyde
RANKL: Receptor activator for nuclear factor-kB
TEM: Transmission electron microscopy
TRAP: Tartrate resistant acid phosphatase
